# Psychological and Behavior Changes of Consumer Preferences During COVID-19 Pandemic Times: An Application of GLM Regression Model

**DOI:** 10.3389/fpsyg.2022.879368

**Published:** 2022-04-29

**Authors:** Larisa Ivascu, Aura Emanuela Domil, Alin Emanuel Artene, Oana Bogdan, Valentin Burcă, Codruta Pavel

**Affiliations:** ^1^Faculty of Management in Production and Transportation, Politehnica University of Timisoara, Timioara, Romania; ^2^Academy of Romanian Scientists, Bucharest, Romania; ^3^Faculty of Economics and Business Administration, West University of Timisoara, Timisoara, Romania

**Keywords:** e-commerce, consumer psychology, online acquisitions, consumer behavior changes, COVID-19 pandemic

## Abstract

The life we considered normal was disrupted due to measures taken to limit the spread of the novel coronavirus. Quarantine, isolation, social distancing, and community containment have influenced consumer behavior and contributed to the rapid development of e-commerce. In pandemic times, even those unfamiliar with the online environment have had to adapt and make acquisitions in this new manner. Hence, we focused our research on measuring the perception of consumers on how the restrictive measures imposed to limit the spread of the COVID-19 virus had influenced their decision to buy a product or service from the online environment, given that purchases are highly subjective and influenced by cumulative effects of economic, social, psychological and behavioral factors. Our paper comes with additional insights from the literature. It adds empirical evidence that reveals that the number of transactions and the value per transaction increased during the COVID-19 pandemic and highlights that online purchases will continue as such even after the pandemic.

## Introduction

Since 2020, the world has been affected by the emergence of SARS-CoV 2, a novel coronavirus that has unbalanced global economies with an impact unprecedented since the Great Recession (UN, 2020). On 11 March 2020, the WHO classified COVID-19 as a pandemic and imposed measures taken to limit the spread, like social distancing and quarantining or closing areas of activity where the virus cannot be controlled (WHO, 2020).

Daily activities could be carried out through online platforms, namely remote working, online courses, or online purchases. Thus, digital technologies played an important role in humanity's “new normal” in a pandemic context. The COVID-19 crisis has forced the development of digitalization which has significantly also affected the growth of e-commerce, a highly impacted segment due to repeated lockdowns and constant market fluctuations that have urged consumers to purchase more through online marketplaces (Sarfraz et al., 2019; Alessa et al., 2021; Fedushko and Ustyianovych, 2022).

However, the pandemic context has generated changes in consumers' behavior. On the one hand, at the beginning of the pandemic period, consumers shifted toward health and safety while maintaining a preference for inexpensive goods and services, given the economic crisis expected to occur alongside the health crisis (Abdullah et al., 2018; Guthrie et al., 2021). On the other hand, because most of the activities were transposed in the online environment, in the long run, given the epidemiological waves and high incidence, consumers have focused on products that bring comfort and a sense of coziness to a living space that became, almost overnight, work environment as well (Ajaz et al., 2020; Gu et al., 2021).

Our aim in this paper is to bring some insights regarding current trends in e-commerce in pandemic times like the ones generated by the emergence of the novel coronavirus disease. In this context, we focused our research on measuring consumer perception of how the restrictive measures imposed to limit the spread of the COVID-19 virus had influenced their decision to buy a product or service from the online environment, given that purchases are highly subjective and influenced by cumulative effects of economic, social, psychological, and behavioral factors. Our research investigated the main factors, difficulties, and advantages of making online acquisitions under COVID-19 pandemic restrictive measures through a questionnaire distributed online. We have used a five-point Likert scale consisting of questions that address different elements that influence the decision to make e-commerce transactions.

In this manner, we tried to get an updated picture of how e-commerce is perceived by consumers in the “new normal” of today, as traditionally, there have been claimed several systemic issues that affect the quality of online purchases, such as uncertainty for the quality of the product, the trust in the supplier, shipping problems, limited delivery or ordering time, and access to product information (Hanus, 2016; Shah et al., 2019).

Our paper comes with additional insights within the literature. It adds empirical evidence highlighting that the number of transactions and the value per transaction increased during the COVID-19 pandemic. Also, our study results reveal that online purchases will continue so even after the pandemic period.

The proposed research is structured in five sections. The first section, the present one, highlights the preliminary aspects of the scientific approach. The second section shows the background and the relevant scientific literature, and the next two sections present, respectively, the research methodologyand a discussion of the results obtained. Finally, the fifth section concludes our undertaken case study.

## Literature Review

The pandemic caused by the emergence of the novel coronavirus disease has substantially changed the life we considered normal and brought, almost overnight, national health systems close to collapse. Humanity has been forced to adapt to face this global challenge (Baker et al., 2020). Hence, to limit the rapid spread of the virus, worldwide governments have imposed restrictive measures ranging from bans of large events, school and university closures, remote working, to a temporary shutdown of the economy, highlighting the urgent need for a strategic digital transformation (EU, 2020).

Most of the daily activities were transposed into the online environment in countries that imposed restriction measures. These measures have had a major impact on the e-commerce segment too, which registered a real breakthrough due to the closure of physical retail stores. Hence, in times of uncertainty, online shopping had become the most accessible option for consumers to satisfy their consumption needs, creating at the same time tremendous pressure on suppliers of essential goods, such as pharmacies and grocery stores, to keep up with the growing demand (Koch et al., 2020).

However, e-commerce registered a spectacular breakthrough over time, and the pandemic generated by the novel coronavirus has amplified this trend. According to the COVID-19 and e-Commerce: A Global Review Report, the global retail market share increased from 14% in 2019 to 17% in 2020. Also, according to the same research, digital marketplaces and e-commerce platforms recorded an increase in transactions from 5% to over 100% in 2020 compared to last year (Sirimanne, 2021).

Data provided by Eurostat, based on the results of a survey carried out in 2021 on ICT (Eurostat, 2021), highlights that the share of e-customers among internet users is growing. Between 2016 and 2021, the largest increase in e-commerce was recorded in the Czech Republic, Hungary, Romania, Slovenia, Croatia, and Lithuania, with most purchases being made by e-shoppers aged between 16 and 24, closely followed by the age group 25–54 (Eurostat, 2021). On the one hand, the main advantages of e-commerce contributed significantly to this growth; namely the convenience of being able to shop anytime and anywhere, access to a broader range of products, and the possibility to easily compare prices and view reviews from other consumers. On the other hand, the restrictive measures imposed to limit the spread of the virus also contributed to the growing trend.

According to Eurostat, in new shopping patterns, e-commerce purchases are influenced by gender, age, level of education, and employment situation. Hence, the share of male online shoppers among internet users is slightly higher than for women. Also, the economic volatility of present determines that consumers to pay more attention to their finances, with repercussions on purchasing power (Abdullah et al., 2021; Cosmulese et al., 2021). Still, studies reveal that customers with a higher level of education tend to purchase more (Eurostat, 2021).

Before COVID-19, most online acquisitions involved the purchase of clothes, shoes, or accessories. In the web era, tourism, fashion, luxury goods, and the organic sector focused on consumption (Zhang, 2021). But, the emergence of the novel coronavirus influenced consumer behavior and preferences (Koch et al., 2020). Hence, declaring COVID-19 as a pandemic led to panic buying of goods of strict necessity, including medication, antiseptics, and disinfectants (Loxton et al., 2020; Shehzad et al., 2020; Rai, 2021), with researchers proving that a consistent supply of goods creates a sense of security for consumers in uncertain times (Prentice et al., 2020). The fear of infection with a virus about which not much was known, and physical store closures, determined the increase in online shopping (Mason et al., 2020). The COVID-19 pandemic appears to be a significant acceleration factor in the e-commerce segment (Pollák et al., 2021).

Hence, the ongoing uncertainty created by the pandemic context changed the consumption patterns (Kirk and Rifkin, 2020) and buying decisions (Mason et al., 2020). The restrictive measures imposed have limited social life and pushed consumers to shop online so that, during the pandemic period, socialization took place in the virtual environment, a channel used now by shoppers also as a tool to identify products, collect information, evaluate similar goods, and finally make the proper acquisitions of the products that best meet their needs (Lv et al., 2020). It seems that customers, due to the pandemic period, ordered online more often than normal, became more experienced, and their awareness has increased (Gu et al., 2021). Also, they became more selective and shifted to local brands for their acquisitions (Sumarliah et al., 2021). Studies state that the pandemic generated an increase in the sales of medical supplies, sporting items, children's products, and entertainment goods, alongside the growth of food sales (Király et al., 2020).

Due to the period of uncertainty, researchers depicted that the percentage of spontaneous purchases decreased, and the percentage of planned purchases increased (Eger et al., 2021). Is this behavior driven by hedonic or by utilitarian motives? Online acquisitions in uncertain times are motivated by their usefulness or by the entertainment and enjoyment experience the product or service provides to consumers. There are studies such as those conducted by Koch et al. (2020) who reveal that hedonic motivation is a better predictor of purchase intentions than utilitarian motives in pandemic times, consumers being able to prioritize essential products for their wellbeing, and de-prioritized products that are not necessary (Kurtisi and Alver, 2021). This result is also expected because, in times of uncertainty, the consumer is tempted to buy, mainly, what is valuable and necessary. Also, the aforementioned research highlights that women and individuals practicing social distancing show higher levels of hedonic motivation.

**Figure 1:**
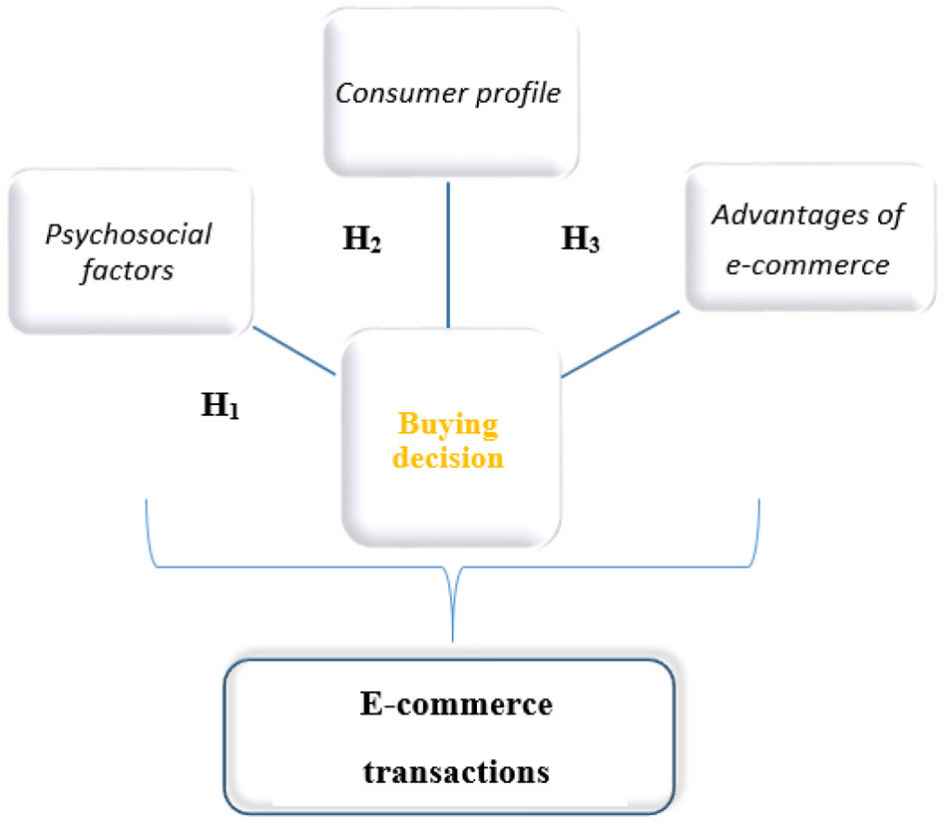
Hypotheses development. Source: authors' own projection.

With these aspects under consideration, this research study proposes the following main hypotheses, depicted in Figure 1:

**H1:** *Psychosocial factors influence the decision to conduct e-commerce transactions*.
**H2:** *The consumer profile influences the decision to carry out e-commerce transactions*.
**H3**: A *good awareness of the advantages of e-commerce transactions influences such transactions*.

Some lessons were learned from this crisis, but consumer behavior cannot always be predicted in uncertain times. In this context, we consider that the way the online shopping system is perceived depends mainly on the interest given by companies in adapting their strategies to the new digital environment. Suitable applications to order online, quality products, and timely delivery represent the primary conditions necessary for the development of online commerce even after the pandemic period. Also, from our point of view, the efficiency of e-commerce depends mainly on the consumer's interests, motivation, and habits.

## Methodology

### Data Collection

**Figure 2:**
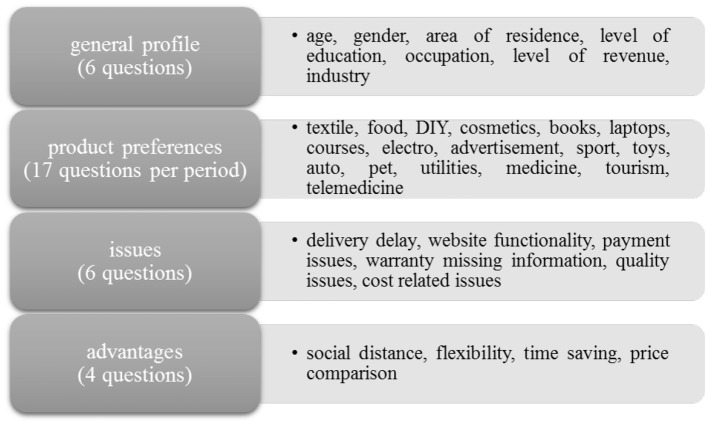
Topics addressed within the questionnaire disseminated. Source: authors' projection.

The research method considered in our study is a questionnaire which allows us to gather information about consumers' perceptions of e-commerce transactions. As the decision to buy a product or service is highly subjective and influenced by cumulative effects of economic, social, psychological, and behavioral factors, the questionnaire is designed to address all those dimensions. The questionnaire consists of 78 closed questions, considering a five-point Likert scale, which addresses different elements of the decision to make e-commerce transactions, as illustrated in Figure 2. The questions addressing the consumers' perception of factors, difficulties, and advantages of e-commerce are also specified in detail, as they are the basis for the design and the estimation of the latent variables (constructs) we intend to estimate in the study, to reduce the data and simplify the econometric model to be estimated. Questions concern both periods analyzed, respectively, the period prior COVID-19 pandemic and the period after the COVID-19 pandemic started.

To check the relevance of public policies in supporting the transition to the home-office regime, we are checking through a set of three questions whether the subsidies granted are used to cover the expenses for e-commerce transactions value.

Additionally, we include five questions that provide us with the dependent variables in our study. We consider the value per transaction estimated on a monthly average and monthly estimated number of e-commerce transactions in each period analyzed. Both variables consist of interval type of data. Another dependent variable in our study relates to consumers' opinion on the likelihood they will perform on future e-commerce transactions, which is measured considering a five-point Likert scale.

Items selection to design our questionnaire has focused mainly on already well-known drivers for consumers' decision to buy, as Kalia et al. (2016) reviewed. However, the definition of consumers' profiles is similar to Zhang (2021). Further, shopping experience and consumer preferences definition have similar reasoning to Vijay et al. (2018). Nonetheless, to adjust the initial design of the questionnaire to the current context of the COVID-19 pandemic, we refer to the approach of Rao et al. (2021) and Hesham et al. (2021), especially concerning the perceived risk and uncertainty of e-commerce buying decisions.

A total number of 334 replies have been received. Dissemination of the questionnaire was available for ~3 months, respectively, the period May–July 2021. Most of the replies are from female respondents, as about 66.35% of respondents were women. More than 68.25% of the respondents are young people between 18 and 30 years, followed by a percentage of 17.14% of respondents with ages between 30 and 40 years. A total of 69.84% of respondents live in an urban area. Related to the level of revenue, our sample is balanced between groups, as the highest weight of a group is related to the respondent having revenue per month of <1,500 lei, summing up to about 36.51%. It is followed by respondents with a level of revenue between 1,500 and 2,500 lei, gathering ~20.32% of our sample. The respondents with a level of revenue between 2,500 and 3,500 lei cover about 19.68% of the sample. From the perspective of their occupation, the respondents are mainly students with a weight of 18.8% in our sample, while the second major group is represented by employees who count for 45.51% of the sample.

### Correspondence Analysis

Evaluation of the structure of the relationship between categories of categorical variables is best reflected through a correspondence analysis. Starting from the contingency table describing the frequency of each association of categories of the variables analyzed, we reach a transformed set of data, summing up a correspondence matrix. To measure the relationship between the row profiles and the column profiles from the correspondence matrix, it is measured the distances between rows and columns using the χ^2^ metric, considering the relations below (Barczak et al., 2021):

- for rows, the formula is:

(1) $$\begin{array}{r} \chi^{2}=d^{2}\left(h,h^{\prime }\right)=\underset{j}{\sum }\frac{{\left(p_{hj}/p_{h}-p_{h^{\prime }j}/p_{h^{\prime }}\;\right)}^{2}}{p_{j}} \end{array}$$

where: *d*^2^*(h,h')* is the distance between the *h*-th and *h*'-th row, *p*_*hj*_/*p*_*h*_ are the row profile elements (also called masses in correspondence analysis theoretical framework), while *p*_*j*_ are the average row profile elements, with h,h' = 1,2,…,H̃.

- for columns the formula is:

(2) $$\begin{array}{r} \chi^{2}=d^{2}\left(j,j^{\prime }\right)=\underset{j}{\sum }\frac{{\left(p_{hj}/p_{j}-p_{hj^{\prime }}/p_{j^{\prime }}\;\right)}^{2}}{p_{h}} \end{array}$$

where: *d*^2^*(j,j')* is the distance between the *j*-th and *j*'-th row, *p*_*hj*_/*p*_*j*_ are the column profile elements, while *p*_*h*_ are the average column profile elements, with *j,j*' = 1,2,…,*J*.

Our study's central element of analysis is the total inertia, which can be defined as a measure of variance in row profiles. These column profiles show how far the row profiles (column profiles) are from their average profile. Once determined the *n*- dimensional space that best represents the points derived from the data is collected, the resulting configuration is rotated to maximize the variance explained by each dimension. The inertia is determined as a weighted average of the distance χ^2^ between the columns and the rows profiles (Greenacre, 2007; Barczak et al., 2021):

- for rows, the formula is:

(3) $$\begin{array}{r} \emptyset_{h}^{2}=\underset{h}{\sum }r_{h}\cdot d_{h}^{2} \end{array}$$

where: $d_{h}^{2}$ is the distance between *h*-th row and the corresponding centroid, while *r*_*h*_ is the sum of frequencies in the row of the correspondence matrix (mass of the row);

- for columns, the formula is:

(4) $$\begin{array}{r} \emptyset_{j}^{2}=\underset{h}{\sum }c_{j}\cdot d_{j}^{2} \end{array}$$

where: $d_{j}^{2}$ is the distance between *j*-th column and the corresponding centroid, while *r*_*j*_ is the sum of frequencies in the column of the correspondence matrix (mass of the column). Starting from the presumption that the row (column) average profile (centroid) supports the hypothesis of homogeneity, the total inertia is determined as $\frac{\chi^{2}}{n}$, explain the level of heterogeneity that is not explained by the sample size.

### Data Reduction

As our sample consists of many questions and as many of them relate to similar core concepts, such as consumers' preferences, we want to reduce the data collected to ensure simplicity and clarity for the econometric model that is later estimated. For this purpose, we proceed to categorical principal components analysis (CatPCA), which is specially designed for categorical data collected through questionnaires (Blasius and Theessen, 2012). Categorical principal components analysis is proper to reduce ordinal data, transforming it into numerical scale by using an estimated non-linear non-monotonic function of transformation (Meulman et al., 2004). The method consists of optimal scaling of categorical data by assigning optimal scale values to each category. The overall variance accounted for the transformed variables is maximized for a specified number of dimensions.

Suppose we have *n* individuals for which we have collected scores for *m* questionnaire items. Data collected is represented in an *S* matrix where *x*_*ij*_ represent the score for individual *i* for item *j*. Those object scores are restricted by relation *S*^*T*^ · *S* = *n* · *I*, where *I* is the identity matrix. Object scores are centered values as well, as they are subject to restriction 1^*T*^ · *S* = 0, where 1^*T*^ is the vector of ones. Each ordinal data *x*_*ij*_ is transformed into a quantified value, based on the scale dimension established prior to the analysis, based on the theoretical framework, using a function of transformation φ. Quantified scores *q*_*ij*_ = φ(*x*_*ij*_) are standardized, considering the restriction ${q_{j}^{T}\cdot q}_{j}=n$. Those scores are multiplied by a set of optimal weights which are called component loadings. The matrix of component loadings *A* consists of *m* rows, like the number of items on the questionnaire, and *p* columns representing the number of components/dimensions identified.

Maximization of variance accounted for the transformed scores consists of minimizing the loss function (5) that measures the difference between original data and principal components, expressed by the function above, using an alternative least squares algorithm (Linting and van der Kooij, 2012).

(5) $$\begin{array}{r} L\left(Q,A,S\right)=\frac{1}{n}\cdot \overset{m}{\underset{j=1}{\sum }}tr{ace\left(q_{j}\cdot a_{j}^{T}-S\right)}^{T}\cdot \left(q_{j}\cdot a_{j}^{T}-S\right) \end{array}$$

The method of CatPCA is performed considering a rotated solution, obtained through the Varimax rotation procedure. After simulation of CatPCA solutions for different dimensions, we remain to the solution that maximizes the variance factors accounted for in the sample, but with a minimum number of factors, to reduce the risk of components overlapping (Hair et al., 2019). The procedure is performed separately for both periods analyzed in this study.

### Ordinal Regression Analysis

Once estimated the scores for each construct, we proceed to an econometric analysis to assess their marginal effect on consumers' decisions concerning the value per e-commerce transaction and on consumers' opinions regarding the likelihood to perform future e-commerce transactions. For this purpose, we estimate a generalized ordinal regression model, as the dependent variable indicates clear order between the different possible values (Garson, 2021). The dependent variable in the case of each model estimated is an ordinal type variable that can take five different levels, depending on:

- the levels of Likert scale used to measure consumers' opinion on the likelihood they will make purchases online even after the COVID-19 pandemic ends;
- the mean of intervals considered defining the number of transactions made online per month, and the value per transaction made online on a monthly average.

In each estimated model, we consider the minimum value possible as a reference. In general, in the case of *k* possible values for the dependent variable, we estimate *k* − 1 binary regression models expressed by the relation below:

(6) $$\begin{array}{r} \ln\frac{P\left(y_{i}=j\right)}{P\left(y_{i}=r\right)}=\beta_{j}\cdot x_{i} \end{array}$$

where β_*j*_ is the vector of regression coefficients, *P*(*y*_*i*_ = *j*) is the probability that outcome *j* is selected, *r* is the “pivot” (reference) outcome, whereas x_i_ represents the vector of independent variables considered for each respondent included in our sample, including the constructs determined based on the CATPCA procedure and variables describing respondent's profile relevant characteristics for the analysis.

Based on this odds ratio, we determine the probability that an individual changes his preference, from the *r* outcome to the new *j* preference, based on the relation below:

(7) $$\begin{array}{r} P\left(y_{i}=j\right)=\frac{e^{\beta_{j}\cdot x_{i}}}{1+\sum_{t=1}^{k-1}e^{\beta_{j}\cdot x_{i}}} \end{array}$$

Additionally, we can determine the odds ratio determined by the ratio $\frac{P\left(y_{i}=j\right)}{1-P\left(y_{i}=j\right)}$ that show the chance that a respondent changes his initial option.

## Results and Discussions

### Descriptive Statistics

The analysis will follow the section with a comparative approach of variables analyzed to identify significant differences in consumers' purchasing behavior in the context of the COVID-19 pandemic and its short-term effects.

Table 1 provides the descriptive statistics on the number of transactions and the average value per transaction, as estimated by respondents during questionnaire dissemination. Looking at the mean of each panel, we observe no significant differences, as prior to the COVID-19 pandemic ~3.078 transactions of about 395.9 Ron were made on average, compared with the period during the COVID-19 pandemic, characterized by a mean of 3.648 transactions of about 488.4 Ron, which is slightly higher, a result similar to the report provided by Eurostat (2021). Looking at the standard deviation of both variables analyzed, for each period considered for analysis, we observe a relatively high variation among our sample, which indicates a relatively heterogeneous sample in terms of the number of online purchasing transactions and value per transaction.

**Table 1:** Descriptive statics purchases.

**Period**		**Prior COVID-19 pandemic**		**During COVID-19 pandemic**		**Opinion on future e-commerce transactions**
		**Number transactions**	**Value transactions**	**Number transactions**	**Value transactions**	
Mean		3.078	395.9	3.648	488.4	3.662
Median		1.50	350	4	350.0	4
Mode		1.50	350	1.50	350.0	4
Std. Dev.		2.457	388.8	2.389	420.6	1.100
Skewness		0.852	1.739	0.651	1.322	
Kurtosis		−0.650	2.513	−1.091	0.920	
Percentiles	25	1.500	100	1.500	100.0	3
	50	1.500	350	4	350.0	4
	75	4	350	4	750.0	4.250

*Source: authors' calculation*.

However, the polarization of options for e-commerce seems to be deepened. Despite the same median value of 4 transactions performed, at about 350 Ron for both periods, the third quartile related to the period during COVID-19 pandemic of 4 transactions translated into ~750 Ron average, is significantly higher, compared with the period before the COVID-19 pandemic, in terms of value per transaction. Therefore, the increase in value per transaction is mainly derived from the increase of value per transaction of some respondents who expended high amounts on online purchases. Our results are similar to those obtained by Sirimanne (2021).

Respondents considered whether they would continue with online purchases, we observe that most of them believe that they will continue, as they provide a median rating of 4 (“agree”), related to a standard deviation of only 1.1. Instead, the results show they continue to be reserved on how they perceive e-commerce, looking at the 3rd quartile (4.25), because of various factors that we will further analyze in this section, such as the trust in the trader, available payment tools, price considerations or even lack of information on products' warranty. It seems that these disadvantages identified in our study are similar to those obtained by Hanus (2016).

### Consumers' Purchasing Behavior in the Presence of Subsidies

The state and some companies have decided to provide either some tax incentives or even some small financial support for employees who had to transition to the home-office regime. In Table 2, we check if there is a statistical impact on the increase of e-commerce volumes of those financial support schemes.

**Table 2:** Descriptive statics purchases.

**Distribution**		**Increase in e-commerce perceived**				
		**Totally disagree**	**Disagree**	**Neutral**	**Agree**	**Totally agree**
Home-Office acquisitions	No	51	32	28	9	3
	Yes	54	45	67	27	18
**Statistics testing influence**	**Statistic**		**Value**		**df**	**Asymptotic significance (2-sided)**
	Pearson chi-square		15.925		4	0.003
	Likelihood ratio		16.629		4	0.002
	Linear-by-Linear association		15.572		1	0.000

*Source: authors' calculation*.

The results of each statistical test performed show that respondents perceived those schemes as relevant for the increase in e-commerce, especially in the case of respondents that have declared they have worked home-office during the current COVID-19 pandemic, thus having to ensure an environment conducive to work from home, like Gu et al. (2021) depicted. However, those results do not offer insights into consumers' preferences, especially during the COVID-19 pandemic. This information is essential as the financial support schemes mainly addressed the need for IT equipment to facilitate the transition to a home-office working regime. Further in the section, we check if there are significant changes in consumers' preferences when discussing e-commerce.

### Changes in Consumers' Preferences

We have included a dedicated section on the questionnaire disseminated, consisting of 17 items aimed to measure consumers' preferences for different types of products available on the market. Despite the insignificant changes noted in consumers' behavior in the context of the COVID-19 pandemic (Zhang, 2021), we want to get some insights into the dynamics of consumers' preferences concerning specific product types, including more specific ones such as telemedicine services. For this purpose, similar to Valaskova, 2021, we have proceeded to a correspondence analysis to draw an image of consumers' preferences, separately for the period prior to the COVID-19 pandemic and, respectively, for the period during the COVID-19 pandemic.

In Table 3 we summarize the inertia statistics, specific for the analysis of heterogeneity in consumers' preferences (Greenacre, 2007). As inertia is a measure of the variation among the sample analyzed, which does not depend on sample size, we expect to have lower values in case of more homogenous preferences among consumers analyzed. The results show that the first dimension in our correspondence analysis describes most inertia. The results on the level of inertia per dimension extracted are explainable, as the second dimension describes better consumers' preferences only for the area of DIY (0.781), household electronics (0.744), laptops, tablets, and other similar devices (0.554). Those results suggest that consumers' preferences are more homogenous in the areas characterized by higher prices, additional technical knowledge requirements for purchasing, warranty concerns, etc.

**Table 3:** Statistics on correspondence analysis related to product type choice.

**Panel**	**Prior COVID-19 pandemic**			**During COVID-19 pandemic**		
**Dimension**	**Singular value**	**Inertia**	**Proportion of inertia %**	**Singular value**	**Inertia**	**Proportion of inertia %**
1	0.365	0.133	0.818	0.359	0.129	0.817
2	0.158	0.025	0.153	0.151	0.023	0.145
Total		0.163	1.000		0.158	1.000
Chi Square	924.42		895.254			
Sig.	0.000^a^		0.000^a^			

a *64 degrees of freedom*.

The results show highest values of inertia on items level are identified in the case of utilities (0.059), textiles (0.022), and telemedicine (0.021), in line with the results obtained by Guthrie et al. (2021), while the lowest values relate to sports (0.001) or tourism (0.001). Therefore, it seems that on the online e-commerce platforms, a high homogeneity is found in advertisement, sports, and tourism, which are expected as those kinds of products are mainly acquired *via* online e-commerce platforms.

In Table 4 we illustrate the product types with the TOP 3 highest and TOP 3 lowest differences in change in the inertia statistic. This way, we show which product types have suffered the highest changes in consumers' preferences to buy online in the context of the COVID-19 pandemic. First, we observe that the highest positive difference is related to the services of utilities, which suggests that heterogeneity in the sample increases, meaning that consumers' preferences to purchase this type of service vary significantly among the respondents. An essential root cause can be the lack or insufficient knowledge of recent information technologies. Second, we observe that the decrease in the measure of inertia related to online medical services suggests the current pandemic context forced consumers to have a more favorable position on purchasing such services online. Until recently, consumers have shown reluctance to acquire such services online. This change in consumer preferences has put a lot of pressure on suppliers of essential goods, such as pharmacies and grocery stores, to keep up with the growing demand, as Koch et al. (2020) depicted.

**Table 4:** Inertia statistics on TOP 3 highest and lowest values.

**Product type**	**Inertia**		**Contribution of point to inertia**				**Differences on inertia**
			**Dim. 1**		**Dim. 2**		
**Period**	**Prior COVID-19 pandemic**	**During COVID-19 pandemic**	**Prior COVID-19 pandemic**	**During COVID-19 pandemic**	**Prior COVID-19 pandemic**	**During COVID-19 pandemic**	
Utilities	0.059	0.067	0.819	0.883	0.178	0.117	0.008
Textile	0.022	0.025	0.932	0.958	0.052	0.037	0.002
Sport	0.001	0.003	0.233	0.427	0.253	0.457	0.002
Food	0.003	0.001	0.852	0.803	0.046	0.052	−0.002
DIY	0.005	0.002	0.115	0.440	0.781	0.361	−0.003
Telemedicine	0.021	0.016	0.839	0.796	0.155	0.194	−0.005

*Source: authors' calculation*.

**Figure 3:**
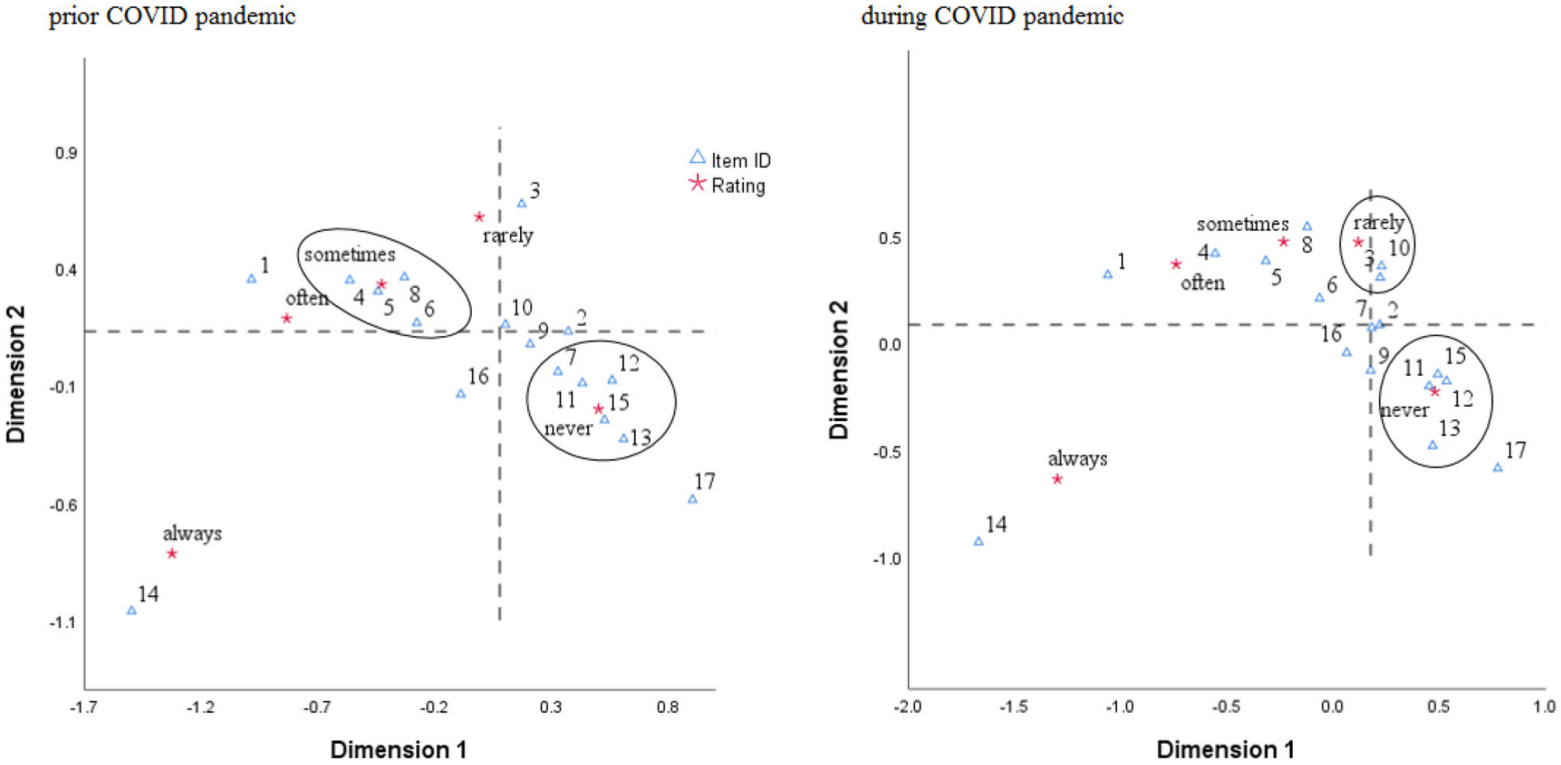
The pattern of consumers' behavior based on correspondence analysis. Source: authors' calculation. Product types are coded as follows: 1, textile; 2, food; 3, DIY; 4, cosmetics; 5, books; 6, laptops; 7, courses; 8, electro; 9, advertisement; 10, sports; 11, toys; 12, auto; 13, pets; 14, utilities; 15, medicine; 16, tourism; 17, telemedicine.

In Figure 3, we better illustrate the changes in preferences, this time related to the rating given by respondents as a measure of the likelihood they would buy the respective product type online. On the one hand, a slight difference between the two periods analyzed is observable only in the case of online courses (coded as “7”), which seem to be day by day a feasible alternative tool for traditional and continuous education. Platforms such as Coursera, or free online training available on the internet, are widely spread on consumers' preferences, providing high-quality courses, positively impacting consumers' performance. On the other hand, the sports product type seems to change slightly negatively compared to prior to the COVID-19 pandemic. Consumers prefer to purchase those products traditionally and only rarely through the online platform or dedicated websites.

In Table 5, we provide a summarized picture of consumers' preferences from the perspective of changes in the measure of inertia, starting from ratings provided by respondents to the questionnaire as a measure of likelihood to buy online products and services.

**Table 5:** Inertia statistics on consumers' ratings concerning likelihood of purchasing online.

**Product type**	**Inertia**		**Contribution of point to inertia**				**Differences on inertia**
			**Dim. 1**		**Dim. 2**		
**Period**	**Prior COVID-19 pandemic**	**During COVID-19 pandemic**	**Prior COVID-19 pandemic**	**During COVID-19 pandemic**	**Prior COVID-19 pandemic**	**During COVID-19 pandemic**	
Always	0.061	0.073	0.852	0.903	0.140	0.092	0.011
Often	0.027	0.024	0.911	0.816	0.020	0.086	−0.003
Rarely	0.011	0.008	0.001	0.095	0.910	0.654	−0.003
Sometimes	0.014	0.010	0.720	0.322	0.188	0.560	−0.004
Never	0.050	0.044	0.934	0.910	0.065	0.087	−0.006

*Source: authors' calculation*.

Overall, the results show a slight change in consumers' preferences in favor of buying products and services online, which is in line with the research conducted by Alessa et al. (2021) and Fedushko and Ustyianovych (2022). Therefore, the current context of the COVID-19 pandemic has determined consumers to reconsider their options toward e-commerce.

However, the results show a slight change toward e-commerce, their reluctance to be visibly present on their options. Further in the study, we analyze the factors that significantly affect their decision on buying products and services through e-commerce channels.

### Reliability Analysis

To analyze the changes in consumers' opinions concerning the main factors influencing their decision to buy online, we first check for our scale used in the design of the questionnaire to be reliable and internally consistent. In Table 6, we provide Cronbach's Alpha statistical test statistics to assess if the Likert scale is reliable and the data collected is relevant for further analysis. Overall, we observe the Cronbach's Alpha statistic for each of the constructs formulated based on the questionnaire design is higher than the minimum threshold of 0.70 (Hair et al., 2019).

**Table 6:** Reliability analysis.

**Variable**	**Factor extracted**	**Panel**	**Period before COVID-19 pandemic**					**Period after COVID-19 pandemic**				
		**Item description**	**Mean**	**St. dev**.	**Cronbach's alpha**	**Loading component**		**Mean**	**St. dev**.	**Cronbach's alpha**	**Loading component**	
**On the decision of purchasing online how important is:**												
Price	Psychological and behavioral factors	The price	3.544	0.29	0.857	0.571		3.601	0.264	0.889	0.405	
Quality		The product quality				0.742					0.613	
Reviews		The forums/reviews				0.433					0.423	
Trust		The trust of site/firm				0.583					0.692	
Payment		The possibility to pay online				0.445					0.670	
Delivery		The delivery time				0.717					0.674	
Ordering		The ease of ordering				0.773					0.661	
Ecological	Other items	The label for ecological product					0.522				0.318	
Brand		The brand				0.309						0.428
Family		The influence from friends/family					0.563					0.637
**Which are the main difficulties encountered by the consumer in online purchasing?**												
Delay	Difficulties	Delay in order delivery	2.426	0.052	0.833	0.475	–	2.426	0.052	0.833	0.475	–
Functional		Website functionality				0.623					0.623	
Payment		Payment system functional				0.610					0.610	
Warranty		Lack of warranty information				0.668					0.668	
Quality		Product quality				0.616					0.616	
Cost		Higher costs				0.557					0.557	
**Which are the main benefits encountered by the consumer on online purchasing?**												
Distance	Advantages	Social distancing	3.863	0.019	0.877	0.617	–	3.863	0.019	0.877	0.617	–
Flexibility		Flexibility				0.847					0.847	
Time		Time savings				0.805					0.805	
Price		Price comparison				0.741					0.741	

*Source: authors' calculation*.

From a scale level perspective, we observe that the highest mean values concern respondents' opinion addressing the measure advantages related to e-commerce influence their decision to buy products and services online (3.863), which suggest a relative impact closer to the 4th Likert scale level, than the neutral scale rating (3). Instead, items addressing difficulties encountered by respondents when buying online seem inconclusive. Therefore, consumers focus instead on the advantages of e-commerce. Difficulties identified are solved either individually or with the support of the website's owners; as with the context of the COVID-19 pandemic, online traders have made visible efforts to improve their customer services.

### Changes in Consumers' Opinion Concerning Factors Influencing Online Purchasing

**Figure 4:**
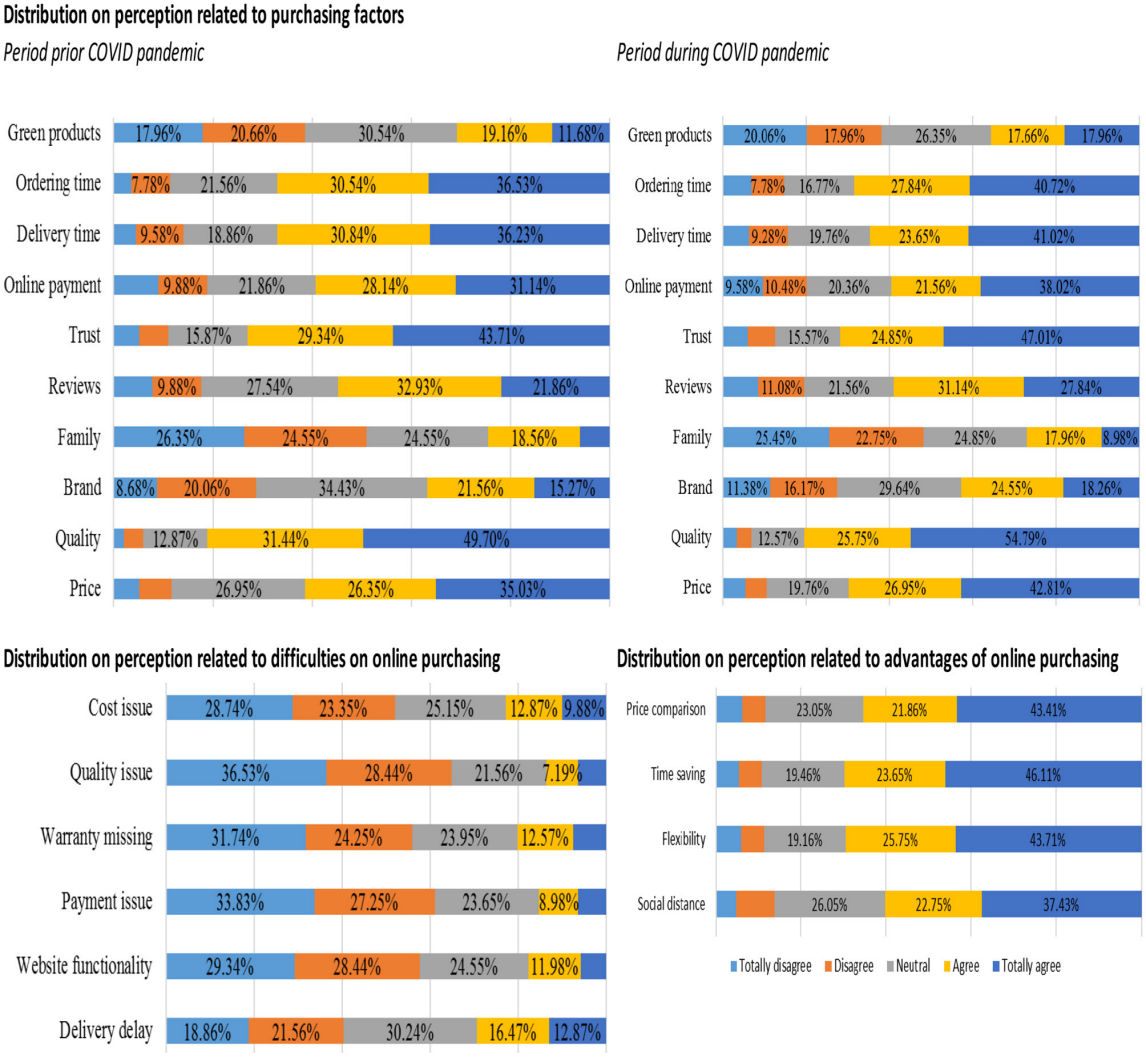
Distributions on items reviewed on the questionnaire. Source: authors' calculation.

In Figure 4, we represent the distribution of probabilities of respondents' ratings on the different items included in the questionnaire, addressing both the factors of the decision to buy online and the related difficulties and advantages.

On the one hand, as expected, we observe consumers' decision to buy products and services online is highly dependent on the quality of products and services and the trust in the traders. The context of the COVID-19 pandemic showed an increase in consumers' opinion on how important factors such as product quality, trust in online traders, product and service pricing, or even delivery time or ordering time are.

On the other hand, we observe that factors such as the influence of family or friends, reviews provided by other customers, consideration of sustainable consumers' preferences, or perception of product and service brand are less relevant for the decision of online buying. One reason could be that consumers can document themselves better in an online environment and with proper customer service support provided through websites that can make their decision easier.

Respondents show high attention to the advantages generated by e-commerce, more attention they pay to the difficulties encountered in deciding to buy online products or services. Developments in customer relations management solutions have provided traders with rigorous, systematic, and fast data access instruments concerning their clients. Moreover, the social platforms provide additional instruments for the traders to capture consumers' preferences using various artificial intelligence tools. Therefore, website owners receive relevant and timely information on consumers' concerns related to their products and customer services. The transition of purchasing decisions in an online environment allows them to collect those data quickly and at low costs.

### Extraction of Reduced Components

Our questionnaire has addressed multiple concerns addressing consumers' rationale, psychological factors, social factors, and economic factors (Peighambari et al., 2016; Deng et al., 2021). For data reduction, we performed a principal components analysis that helped reduce the 20 items disseminated in the questionnaire to only four main components (*constructs*). As our variables consist of categorical data, we proceed to a categorical PCA analysis to ensure the internal consistency of the results (Meulman et al., 2004; Linting and van der Kooij, 2012).

In Table 7, we provide the summary statistics for the components extracted. Those latent variables measure the aggregate effects of items included, capturing the items that present the highest variation among the sample in terms of respondents' rating (Hair et al., 2019). Determined constructs represent the data collected through the questionnaire as they account for more than 66.4% of the variation in the sample for both periods analyzed. The context of the COVID-19 pandemic determined some changes in the representativeness of those constructs, as the factor related to items addressing difficulties encountered during online purchasing (*difficulties*) lose their importance, on the ground of a decrease from 16.03% account for variance in the sample to a percentage of only 11.61%. Instead, most of this decrease is transposed on the increase of variance accounted by psychological and behavioral factors from a percentage of 24.35% to the level of 30.64%.

**Table 7:** Statistics on variation reflected by factors extracted with CATPCA.

**Period**	**Prior COVID-19 pandemic**			**During COVID-19 pandemic**		
**Dimension**	**Cronbach's alpha**	**Eigenvalue**	**% of variance**	**Cronbach's alpha**	**Eigenvalue**	**% of variance**
Psychological and behavioral factors	0.869	4.870	24.350	0.881	6.128	30.640
Other items	0.800	3.663	18.314	0.775	3.791	18.953
Difficulties	0.763	3.206	16.028	0.599	2.322	11.608
Issues	0.707	1.555	7.774	0.044	1.044	5.218
Total	0.973^a^	13.293	66.467	0.973^a^	13.284	66.419

a *Total Cronbach's Alpha is based on the total Eigenvalue. Source: authors' calculation*.

In Table 6, we provide the structure of each construct identified, based on the reference of each construct to the items included on the questionnaire. Based on those results, the initial design of the constructs has not changed significantly. The only change we note is the estimation of a construct (*other items*) that incorporate mainly the item addressing respondents' perception of how they believe their decision to buy online is influenced by feedback received from friends and family members. Despite that these items were expected to be one of the main factors influencing the online purchasing decision, it seems that factors such as. However, the fact that this item covers only a small portion of the variation in the sample shows that feedback from friends and family members is perceived of slightly similar importance for their decision to buy products and services online.

### Constructs Validation

**Figure 5:**
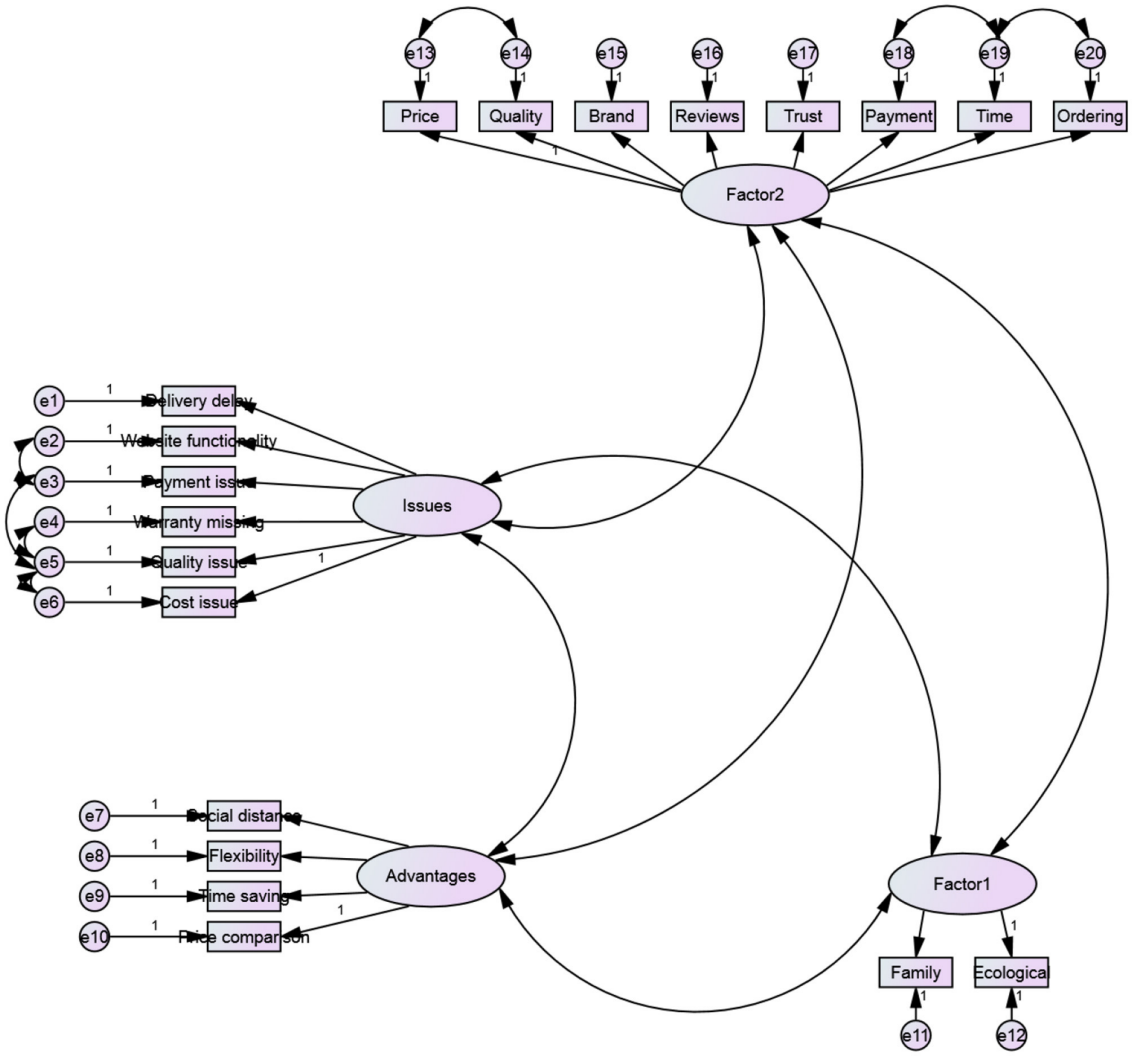
Design of confirmatory factor analysis model. Source: authors' calculation.

Statistical validation of the constructs determined through CATPCA is made by performing a confirmatory factor analysis (DiStefano and Hess, 2005; Blasius and Theessen, 2012; DiStefano et al., 2019). DiStefano et al. (2019) note that the CFA should be estimated with the Asymptotic Distribution Free Estimation method. In Figure 5, we illustrate the design of the confirmatory factor analysis model.

However, as noted by DiStefano and Hess (2005), the use of the maximum likelihood method or the alternative method of asymptotic distribution-free estimation does not generate different results if the categorical variables have an absolute value of skewness and kurtosis lower than 2.

Based on the results provided in Table 8, we can validate the model as statistically significant (Hair et al., 2019). Those results ensure the internal consistency of the model of principal components analysis.

**Table 8:** Statistics on variation reflected by factors extracted with CATPCA.

	**Ideal threshold (Hair et al., 2019)**	**Stat**.	**Stat**.	**Resolution**
CFI	>0.95	0.929	0.947	Moderate
RMSEA	<0.10	0.066	0.06	Moderate
SRMR	<0.07	0.052	0.052	Moderate
Cmin/df	<3	2.44	2.217	Good

*Source: authors' calculation*.

Further, we continue with the evaluation of our CFA model discriminant validity.

In Table 9, we summarize the measure of AVE for each factor extracted and the correlations with the other factors extracted. We observe that the average variance extracted (AVE) is higher than the correlations with the other factors extracted (Hair et al., 2019; Garson, 2021). Therefore, our model proves discriminant validity.

**Table 9:** CFA model discriminant validity.

**Period**	**Factor**	**Other items**	**Issues**	**Advantages**	**Psycho. factors**
Prior COVID-19 pandemic	Other items	**0.727**			
	Issues	0.190	**0.658**		
	Advantages	0.540	0.160	**0.841**	
	Psychological factors	0.659	0.182	0.370	**0.662**
During COVID-19 pandemic	Other items	**0.845**			
	Issues	0.094	**0.669**		
	Advantages	0.553	0.100	**0.933**	
	Psychological factors	0.633	−0.005	0.328	**0.814**

*Source: authors' calculation*.

In Table 10, we provide statistics relevant to assessing the CFA model's convergent validity. The measure of composite reliability (CR) represents a better measure of constructs reliability. The results show that all constructs are reliable as the CR for each extracted factor is higher than the threshold of 0.70 (Garson, 2021).

**Table 10:** CFA internal consistency.

**Factor**	**Cron. Alpha**	**CR**	**AVE**	**MSV**	**Cron. Alpha**	**CR**	**AVE**	**MSV**
Ideal threshold		>0.7	>0.5			>0.7	>0.5	
Other items	0.707	0.898	0.528	0.434	0.044	0.952	0.714	0.401
Issues	0.800	0.820	*0.433*	0.036	0.775	0.828	*0.448*	0.010
Advantages	0.763	0.906	0.708	0.292	0.599	0.964	0.871	0.306
Psychological factors	0.869	0.599	0.438	0.434	0.881	0.792	0.663	0.401

*Source: authors' calculation*.

The measure of average variance extracted (AVE) express how much variation is accounted for by each construct within the variance of items incorporated. Expect for the factor issues for which the AVE measure does not exceed the threshold of 0.50; the rest seem to be sufficiently representative for the items incorporated in the related constructs (Garson, 2021). The concern of convergent validity in case of factor issues suggests that the construct items are not well-correlated. Consequently, we will not consider this construct in further econometric analysis.

### Evaluation of the Marginal Effect on Value per Transaction

Once the design of the construct was estimated, we continue with the final step, respectively, the econometric analysis that evaluates the association between the construct scores estimated before and the average level of value per each online purchasing transaction. In Table 11, we summarize the statistics of the model estimated. The results indicate differences in the determinant of the value per transaction if comparing the two periods of analysis, respectively the period before the COVID-19 pandemic and the period during the COVID-19 pandemic.

**Table 11:** GLM regression model statistics.

**Period**	**Prior COVID-19 pandemic**				**During COVID-19 pandemic**			
**Model**	**(1)**		**(2)**		**(3)**		**(4)**	
	**Coef**.	**Effect**	**Coef**.	**Effect**	**Coef**.	**Effect**	**Coef**.	**Effect**
Psychological and behavioral items	0.103	1.108	0.177	1.194	0.275^**^	1.317	0.389^*^	1.475
	0.117	–	0.122	–	0.114	–	0.121	–
Advantages	−0.067	0.935	−0.098	0.907	0.039	1.039	0.052	1.053
	0.104	–	0.107	–	0.103	–	0.106	–
Other items	0.096	1.101	0.074	1.077	0.198^***^	1.219	0.279^**^	1.321
	0.103	–	0.107	–	0.108	–	0.116	–
**Control variable**								
**Gender**								
Female	–		−0.979^*^	0.376	–		−0.572^**^	0.565
		0.2369	–		0.234			
**Activity**								
Student	–		−2.846^**^	0.058	–		−1.836^**^	0.159
		1.0093	–		0.916	–		
Others	–		−0.665	0.514	–		−1.299^**^	0.273
		0.5793	–		0.589	–		
Employee	–		−0.743	0.476	–		−0.880^***^	0.415
			0.5207	–			0.530	–
**Revenue**								
<1,500	–		−1.652^*^	0.192	–		−0.996^**^	0.369
		0.482	–				0.466	–
1,500–2,500	–		−1.356^**^	0.258	–		−1.375^*^	0.253
		0.429	–				0.417	–
2,500–3,500	–		−1.356^*^	0.258	–		−1.273^*^	0.280
		0.408	–				0.378	–
**Model validation**								
**Omnibus test**								
Chi-Square	–	2.198	–	60.53	–	9.694	–	69.22
Df		3		16		3		16
Sig.		0.532		0.000		0.021		0.000
**Pearson chi-square**								
Value	–	1,220.4	–	1,316.6	–	1,242.4	–	1,159.9
Df		1,189		1,200		1,189		1,200
Value/df		1.026		1.097		1.045		0.967
AIC	–	856.9	–	830.9	–	835.8	–	806.7

*, **, *** *indicates significance at 1, 5, and 10%, respectively. Source: authors' calculation*.

On the one hand, we observe that in the period prior to the COVID-19 pandemic, none of the factors determined a statistically significant effect on the odds that consumers change the value per online buying transaction.

Let us look at the next model estimated, which controls respondents' characteristics. We observe the value per transaction is still not influenced significantly by the constructs based on the questionnaire disseminated.

Instead, we observe that the decision that consumers change the value per online purchasing transaction is statistically conditioned by respondents' characteristics such as gender, activity type, or revenue level. Results show that female consumers are more reluctant to make online purchasing transactions, as they prefer to allocate the lower value of such expenses (*Coef*. = −0.979, *Sig*. < 0.01), compared with male consumers. Therefore, it seems that the value per online purchasing transaction has the odds to increase with a likelihood that decreases with about 0.376 − 1 = −0.624.

Similar results we also observe in case of the marginal effect of the type of activity, or the level of revenue, on the odds consumers would increase the value per online purchasing transaction. Results suggest that students are less likely to increase their value per online purchasing transaction (*Coef*. = −2.846, *Sig*. < 0.05).

The level of revenue also impacts negatively the odds that consumers increase their value per online purchasing transaction, in the case of each level of revenue considered in the analysis, respectively, consumers with revenues <1,500 Ron (*Coef*. = −1.652, *Sig*. < 0.01), with revenue between 1,500 and 2,500 ron (*Coef*. = −1.356, *Sig*. < 0.05), or with revenue between 2,500 and 3,500 Ron (*Coef*. = −1.356, *Sig*. < 0.01). Instead, we observe that higher revenues lead to better odds of the increase in the value per online purchasing transaction, in the case of the psychological level of revenue of 1,500 Ron (0.192 − 1 = −0.808 < 0.258 − 1 = −0.742).

On the other hand, the results of the third model are estimated to show a significant marginal effect of the first and the last factors extracted. However, the construct *psychological and behavioral item*s have a higher impact (*Coef*. = 0.257, *Sig*. < 0.05), compared with the *other items* construct (*Coef*. = 0.198, *Sig*. < 0.10). As noted already from the categorical principal components analysis results, the factors related to feedback received from family and friends are less relevant for the consumers when deciding to increase the value per online purchasing transaction.

Based on results from Table 6, we see that the construct of *psychological and behavioral item*s is mostly influenced by the items addressing consumers' perception of the role of product quality (0.742), ordering time (0.773), and delivery time (0.717), with higher loading that some items which were expected to have higher relevance, such as the price factor (0.571), or the trust on the website (0.583). However, those results do not imply direct implications on consumers' behavior. The principal components analysis better describes the components extracted from the items for which respondents have expressed opinions with higher variance in the sample. Therefore, those results show that items mentioned above are more perceived as mandatory requirements, which are already expected from the consumers to be of a satisfactory level, with the premises of competitive prices, sufficiently diverse offers on the market, and highly exigent national regulation that sanction misconduct such as e-commerce fraud.

Controlling the results for the effect of consumers' profiles, we observe the fourth model estimated that the results increase slightly but determine a similar positive effect on the odds that consumers would increase the value per online purchasing transactions in the future.

Instead, the respondents' profile seems to generate a different impact, as the gender (*Coef*. = −0.572 > −0.979, *Sig*. < 0.05) and the fact that the consumer is a student (*Coef*. = −1.836 > −2.846, *Sig*. < 0.05) have a lower impact on the odds of increasing the value per transaction. A similar negative lower impact on the odds of increasing the value per transaction concerns the different levels of revenue characterizing the consumers' profile.

Therefore, in the context of the COVID-19 pandemic, women have realized the need for e-commerce, even if they were somehow forced by the restrictions implemented to ensure social distance, despite their customs for traditional purchasing behavior, which involves including the need for social interaction during shopping sessions. Similar negative results concern as well-consumers' behavior that is conditioned by the type of activity they perform, but with a lower negative impact for both the employees (*Coef*. = −0.88 > −1.836, *Sig*. < 0.10) and the group of others (*Coef*. = −1.299 > −1.836, *Sig*. < 0.10), compared with the effect valid for the students.

### Evaluation of the Marginal Effect on the Likelihood of Future e-Commerce Transactions

In Table 12, we present the statistics of the econometric model estimated to describe the association between the factors extracted and the option for a future increase in expenses allocated for online purchasing transactions. The period analyzed is this time related to the period during the COVID-19 pandemic.

**Table 12:** GLM regression model statistics.

**Factor**	**Coef**.	**Effect**	**Coef**.	**Effect**
**Model**	**(5)**		**(6)**	
Psychological and behavioral items	0.472^*^	1.604	0.539^*^	1.714
	0.118		0.125	
Advantages	−0.115	0.891	−0.100	0.905
	0.106		0.109	
Other items	0.151	1.163	0.174	1.191
	0.109		0.114	
**Control variable**				
**Area**				
Rural	–	−1.886^***^	0.152	
		0.970	–	
**Activity**				
Student	–	−1.886^***^	0.152	
		0.970	–	
**Revenue**				
2,500–3,500	–	−0.641^***^	0.527	
		0.376	–	
**Model validation**				
Omnibus test				
Chi-Square	–	19.63	–	44.80
df		3		16
Sig.		0.000		0.000
**Pearson chi-square**				
Value	–	1,207.0	–	1,286.7
df		1,189		1,200
Value/df		1.015		1.072
AIC	–	866.7	–	871.1

*, **, *** *indicates significance at 1, 5, and 10%, respectively. Source: authors' calculation*.

As in the case of models estimated in Table 12, the models estimated in Table 12 show a similar impact of respondents' profiles on the odds they would choose in the future to increase their interest in e-commerce. Similar to the other models is the insignificant effect of the construct advantages.

However, the effect of gender on consumers' decision to increase their interest in e-commerce in the future is not statistically significant anymore. It has been confirmed in the case of its role in increasing the odds of an increase in value per transaction. Similar, the only statistically significant effect of the level of revenue is valid only in the case of the consumers with revenues between 2,500 and 3,500 Ron, meaning that only consumers with a higher level of revenue show interest in future e-commerce. The results indirectly suggest that respondents are still reluctant concerning e-commerce, as lower revenue levels do not translate into consumers' favorable opinions concerning future interest in e-commerce.

Overall, the results show that only psychological and behavioral factors influence consumers' decision to increase their interest in e-commerce in the future. The positive impact of constructed *psychological and behavioral items* determines an increase in the odds that future consumers would continue to buy products and services online, even increasing expenses for such purchasing transactions (*Coef*. = 0.472, *Sig*. < 0.01), even after controlling the results for consumers' profile fixed effects (*Coef*. = 0.539, *Sig*. < 0.01). Analyzing together the results in the fourth model estimated and the results in the sixth model, we appreciate that the current context of the COVID-19 pandemic has raised consumers' interest in e-commerce, which is expected to lead to exponentially increasing volumes and values per online purchasing transactions.

Consequently, we can validate hypothesis **H1:** *Psychosocial factors influence the decision to conduct e-commerce transactions*. Only for some groups of respondents, did consumers significantly influence the value per transaction profile characteristics.

Additionally, we see a lower chance that consumers increase their interest in e-commerce in the future (*Coef*. = −1.886, *Sig*. < 0.10), which might be caused either by bad internet connection, reluctance, or the lack of information on the use of recent information technologies, such as the use of online payments. Nonetheless, as long as advertising campaigns do not penetrate enough the potential customers from rural areas, the future of e-commerce is not expected to increase drastically, despite the benefits of time-saving and transport cost savings.

Consequently, we can partially validate the hypothesis **H2:** *The consumer profile influences the decision to carry out e-commerce transactions*, as only for some groups of respondents does the chance in favor of e-commerce increase, based on some consumers' profile characteristics, this time only in case of consumers' area and the type of activity they perform.

The results suggest that we cannot validate the third hypothesis **H3**: *A good awareness of the advantages of e-commerce transactions influences such transactions*, as in all models estimated concerning the chance to change consumers' preferences in favor of e-commerce, regression coefficients are not statistically significant.

## Conclusion

The pandemic period has substantially changed the life we considered normal, and people have learned that to cope with the rules of the “new normal” they must adapt and learn how to socialize, work, and purchase necessary goods in uncertain times.

Digitization played an important role during this period as most activities were transposed online due to the restrictive measures imposed to limit the spread of the novel coronavirus. Alongside digitalization, the e-commerce segment registered a spectacular development because of the fear of infection with a virus about which not much was known and physical store closures. Consumers started to do online shopping. Still, it is interesting to know what influences their buying decisions, namely *Psychosocial factors that influence the decision to conduct e-commerce transactions? Does the consumer profile influence the decision to carry out e-commerce transactions?* A *good awareness of the advantages of e-commerce transactions influences such transactions?* Our paper reveals that consumers' decision to buy products and services online is highly dependent on the quality of products and services and the trust in the traders. The context of the COVID-19 pandemic showed an increase in consumers' opinion on how important factors such as product quality, trust in online traders, product, and service pricing, or even delivery time or ordering time are. Also, the influence of family or friends, reviews provided by other customers, consideration of sustainable consumers' preferences, or perception of product and service brands are less relevant for online buying.

Also, our research highlights that the number of transactions and the value per transaction increased during the COVID-19 pandemic and concludes that online purchases will continue so even after the pandemic.

Hence, in the context of the pandemic generated by the novel coronavirus, e-commerce has become the lifebelt for many traditional stores, which have had to implement or expand online sales quickly, click and collect shopping, or home delivery services. Also, the restrictions have highlighted that access to local services and closer collaboration with local producers are essential to continue the activity even when the traditional supply chain is stopped. Thus, when designing their marketing and product portfolio management strategies and policies, firms must consider the lessons offered by the pandemic that has highlighted that both people and businesses must adapt to the new normal of nowadays if they want to ensure continuity of performance and sustainability over time.

## Data Availability Statement

The raw data supporting the conclusions of this article will be made available by the authors, without undue reservation.

## Ethics Statement

Ethical review and approval was not required for the study on human participants in accordance with the local legislation and institutional requirements. The patients/participants provided their written informed consent to participate in this study.

## Author Contributions

All authors listed have made a substantial, direct, and intellectual contribution to the work and approved it for publication.

## Conflict of Interest

The authors declare that the research was conducted in the absence of any commercial or financial relationships that could be construed as a potential conflict of interest.

## Publisher's Note

All claims expressed in this article are solely those of the authors and do not necessarily represent those of their affiliated organizations, or those of the publisher, the editors and the reviewers. Any product that may be evaluated in this article, or claim that may be made by its manufacturer, is not guaranteed or endorsed by the publisher.
